# Changes E3 ubiquitin protein ligase 1 gene mRNA expression correlated with IgA1 glycosylation in patients with IgA nephropathy

**DOI:** 10.1080/0886022X.2019.1605295

**Published:** 2019-05-06

**Authors:** Youxia Liu, Jie Zheng, Junya Jia, Hongfen Li, Shuiyi Hu, Yujia Lin, Tiekun Yan

**Affiliations:** a Department of Nephrology, Tianjin Medical University General Hospital, Tianjin, PR China;; b Radiology Department, Tianjin Medical University General Hospital, Tianjin, PR China

**Keywords:** Galactose-deficient IgA1, HECW1 mRNA level, rs978056, IgA nephropathy

## Abstract

**Background:** Recent genomewide association study suggested that the top single-nucleotide polymorphism, rs978056, in *HECW1* gene (which encodes HECT, C2 and WW domain containing E3 ubiquitin protein ligase 1) associated with the levels of galactose-deficient IgA1 (Gd-IgA1) in IgA nephropathy (IgAN). However, HECW1 expression in IgAN has not yet been examined.

**Methods:** In the following study, we have enrolled 40 patients with IgAN and 40 healthy controls. The expression level of HECW1, as well as plasma levels of Gd-IgA1 and IgA1, were determined detected.

**Results:** IgAN patients presented with significantly elevated Gd-IgA1 and IgA1 levels compared with those of the healthy controls (*p* < .001 and *p* = .03, respectively). We further divided the patients into two groups according to the median level of HECW1 (0.58). We found the levels of Gd-IgA1 and IgA1 were significantly higher in low HECW1 level group compared with those in high HECW1 level group (*p* = .02 and *p* = .04, respectively). And HECW1 mRNA expression had a significant inverse correlation with Gd-IgA1 levels in IgAN patients (*r*= −0.34, *p* = .03). It seemed that the risk genotype (rs978056 GG) was associated with reduced HECW1 expression in 80 Han Chinese from Beijing, although the difference was not significant (*p* = .09). No significant association with clinical and pathological manifestations was observed between patients with high and low levels of HECW1.

**Conclusion:** We reported for the first time that HECW1 mRNA levels were negatively correlated with Gd-IgA1 levels. Our study points to a new regulatory mechanism of IgAN that can explain the aberrant glycosylation of IgA1.

## Introduction

IgA nephropathy (IgAN), the most common primary glomerulonephritis worldwide, is characterized by mesangial deposition of IgA1-containing immune complexes [1,2]. Approximately 30% to 40% of the patients with IgAN progress to end-stage kidney disease (ESKD) within 20 years after presentation [3]. Multiple studies have established that aberrant glycosylation of IgA1 played an important role in the pathogenesis and progression of IgAN [4–6]. However, limited scientific data regarding the factors and mechanisms that induce the production of galactose-deficient IgA1 (Gd-IgA1) are available.

Association studies found genetic associations between the *C1GALT1, ST6GALNAC2, C1GALT1C1* gene polymorphisms and Gd-IgA1; however, there is a discrepancy on the expression levels of these genes [7–10]. The current progress is in genetic field along with multiple single-nucleotide polymorphisms (SNPs) identified contributed to the production of Gd-IgA1. A recent genomewide association study (GWAS) in IgAN discovered the top SNP, rs978056, in *HECW1* gene (which encodes HECT, C2 and WW domain containing E3 ubiquitin protein ligase 1) associated with the levels of Gd-IgA1 apart from *C1GALT1* and *C1GALT1C1* [11]. HECW1, also known as NEDD4-like ubiquitin protein ligase 1 (NEDL1), has been reported to be expressed in human neuronal tissues and enhances p53-mediated apoptotic cell death [12,13]. However, the association between HECW1 mRNA expression level and Gd-IgA1 levels is still unknown in IgAN.

In the present study, we investigated the HECW1 mRNA expression levels and glycosylation of IgA1 status in patients with IgAN to explore the underlying mechanism of HECW1 in the production of Gd-IgA1.

## Materials and methods

### Subjects

Forty IgAN patients diagnosed in Tianjin Medical University General Hospital from May to October 2017, were enrolled in this study. The diagnosis was based on the deposition of IgA in the glomerular mesangium by immunofluorescence detection, as well as the lack of clinical or serological evidence of other inflammatory conditions, such as Henoch Schoenlein purpura, systemic lupus erythematosus, or diabetes mellitus. At the same time, 40 healthy volunteers whose age and gender matched with patients were recruited. Plasma was collected from all individuals in this study, for patients at the time of renal biopsy. The plasma samples were stored in aliquots at −80 °C for the subsequent use. Clinical information, including 24-h urine protein excretion, blood pressure, and total IgA1 levels, were collected at the time of renal biopsy. The estimated glomerular filtration rate (eGFR) was calculated using the Chronic Kidney Disease Epidemiology Collaboration creatinine equation. The histological lesions were classified according to Oxford classification system.

### Ethics statement

The Medical Ethics Committee of Tianjin medical university general hospital approved the study protocol and informed written consent was obtained from all individuals. All study participants provided written informed consent for the collection of blood samples, and for the examination of clinical records relevant to the study.

### Assay for IgA1 and Gd-IgA1

Total IgA1 and Gd-IgA1 levels in plasma and in cell culture supernatant were determined by ELISA, as previously reported [6]. A standard consisting of native IgA1 purified by normal human plasma (EMD Chemicals, USA) was used as the standard for the quantification of total IgA1. As for Gd-IgA1 detection, the standard consisting of IgA1 protein isolated from plasma of a patient with multiple myeloma using an agarose-bound jacalin affinity chromatography column (Pierce Chemical Company, Illinois, USA). The IgG was removed by a protein G column (GE, Connecticut, USA). And the terminal sialic acid from O-linked GalNAc on bound samples and the standard IgA1 protein were removed by neuraminidase (Roche, Basel, USA), and galactose from O-linked GalNAc was removed by galactosidase (Sigma, Missouri, USA). As the standard IgA1 protein is not entirely devoid of galactose, we expressed the results as U/mL, in which 1 unit of Gd-IgA1 was defined as 1 ng of this standard Gd-IgA1 protein.

### B lymphocytes isolation

About 5 mL venous blood sample was taken into ethylenediaminetetraacetic acid (EDTA) anticoagulated tubes. Peripheral blood mononuclear cells (PBMCs) were separated by density-gradient centrifugation on Ficoll (TBD, Tianjin, China), then washed three times with phosphate buffered saline (PBS) and resuspended in PBS + 1% bovine serum albumin (BSA). Peripheral B lymphocytes were isolated using CD19 positive magnetic beads (Miltenyi Biotec, Cologne, Germany) according to the manufacturer’s instructions.

### RNA extraction and reverse transcription PCR (RT-PCR)

Total cellular RNA was extracted from CD19-positive B lymphocytes using the TRIZOL Reagent (Invitrogen, California, USA). RNA quantity was determined using NanoDrop ND-1000 spectrophotometer. cDNA was synthesized using 300 ng total RNA with revert first-strand cDNA kit according to manufacturer’s protocol (Promega, USA). Resulting cDNA was amplified with a 20 µL reaction mixture using SYBR Green PCR Master Mix (Roche, USA) in an Applied Biosystem 7500 Real-Time PCR System. And the primer pairs of HECW1 and GAPDH were listed in Table 1. The fold changes in between patients and controls were expressed by the 2^−△△CT^ method.

**Table 1.** Primers used to amplify the HECW1 and GAPDH genes.

Gene	Forward Primers (5′-3′)	Reverse Primers (5′-3′)
*HECW1*	CTCCTGCTACAACGGCAACA	TTCTCCTCCTCCTCGTCGTC
*GAPDH*	TTGCCCTCAACGACCACTTT	TGGTCCAGGGGTCTTACTCC

### Association of genotype with gene expression

Genevar software, a database and Java tool designed to integrate multiple datasets, was used to determine associations between sequence variation and gene expression (http://www.sanger.ac.uk/resources/software/genevar/) [14]. The sequence variation and gene expression profiling data were from lymphoblastoid cell lines collected from HapMap3.

### Statistical analysis

For continuous variables, data with a normal distribution were expressed as the mean ± SD and compared by an independent-samples *t*-test, whereas other data were expressed as the median (first quartile and third quartile) and analyzed by the Mann–Whitney U-test. The level of significance was chosen as *p* < .05. All statistical tests were performed using SPSS version 16.0 (Chicago, USA).

## Results

### Baseline clinical characteristics of patients with IgAN

The clinical features of IgAN patients in the present study were shown in Table 2. The levels of IgA1 (1815 μg/ml, [1565 μg/ml, 2583 μg/ml]) in IgAN patients were higher than those of the healthy controls (1397 μg/ml, [840 μg/ml, 1758 μg/ml], *p* = .03). And the grading of the pathological lesions by Oxford classification was shown in Table 2.

**Table 2.** The baseline data for patients with IgAN and healthy controls.

Characters	IgAN (*n* = 40)	Healthy controls (*n* = 40)	*p* value
Male/female	22/18	20/20	.83
Age (mean ± SD, year）	38 ± 11	37 ± 12	.80
SBP (mmHg, mean ± SD)	128 ± 16	125 ± 16	.77
Proteinuria (g/d, median, IQR)	1.23 (0.43–3.84)		
Plasma IgA1 (μg/mL, median, IQR)	1815 (1565–2583)	1397 (840–1758)	.03
eGFR (mL/min/1.73 m^2^)	82.81 ± 25.78		
Oxford classification (%)			
M score (M0/M1)	6 (15)/34 (85)		
E score (E0/E1)	28 (70)/12 (30)		
S score (S0/S1)	22 (55)/18 (45)		
T score (T0/T1/T2)	18 (45)/18 (45)/4 (10)		
C score (C0/C1/C2)	10 (25)/22 (55)/8 (20)		

eGFR: estimated glomerular filtration rate; IQR: interquartile range; SBP: systolic blood pressure; SD: standard deviation.

### mRNA levels of HECW1 in IgAN patients

**Figure 1.**
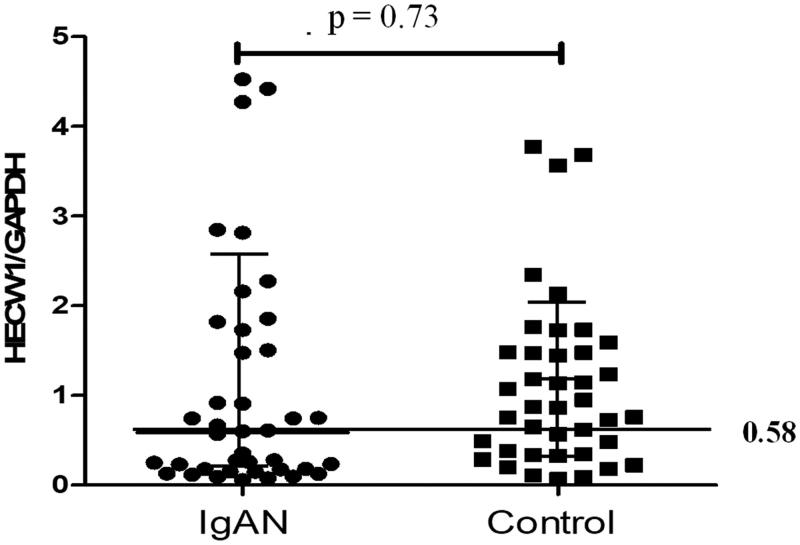
The mRNA expression levels of HECW1 in IgAN patients and healthy controls.

In circulation, CD19^+^ B lymphocytes are the major cells for IgA1 production. Therefore, we isolated CD19^+^ B lymphocytes to detect the different expression of HECW1 mRNA. However, no significant difference were observed between patients with IgAN and controls (0.58 [0.17, 1.67] versus 0.86 [0.35, 1.48], *p*= .73, Figure 1). Data of expression level of HECW1 were non-normally distributed. As shown in Figure 1, some patients with IgAN showed marked low values of HECW1. The median mRNA level of HECW1 (0.58) in IgAN was defined as the cutoff value for dividing samples into two groups, it was shown that the higher percentage of the individuals with IgAN has a low level of HECW1 (50%) comparing with that in controls (35%, *p* = .27). It suggested that more patients with IgAN had low values of HECW1.

### Association between HECW1 expression and Gd-IgA1 levels

**Figure 2.**
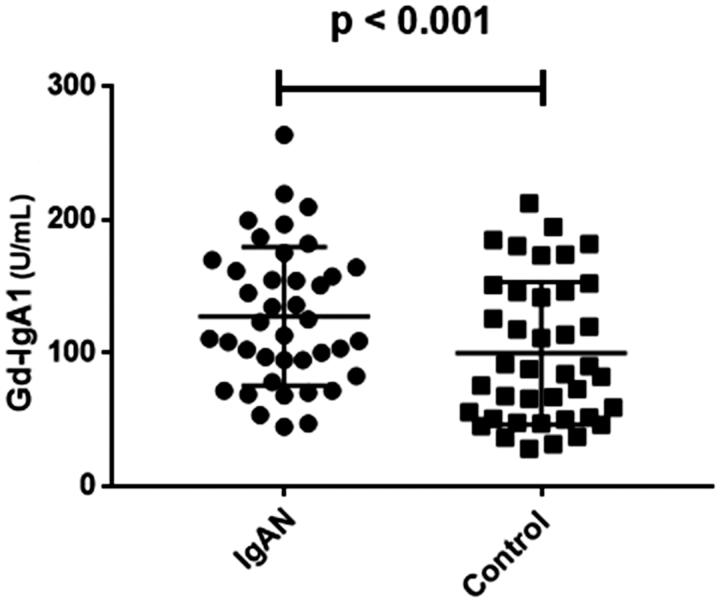
Plasma Gd-IgA1 levels in IgAN patients and healthy control.

**Figure 3.**
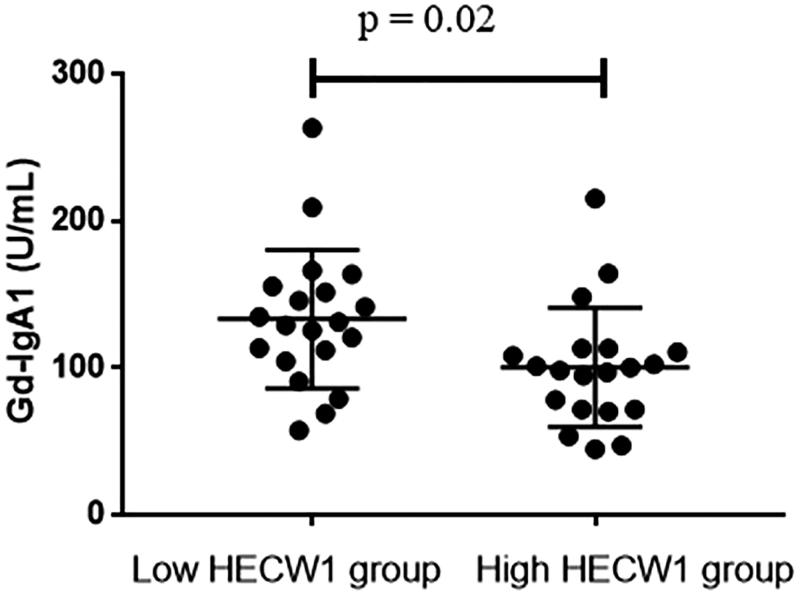
Gd-IgA1 levels in IgAN patients with low HECW1 group and high HECW1 group.

**Figure 4.**
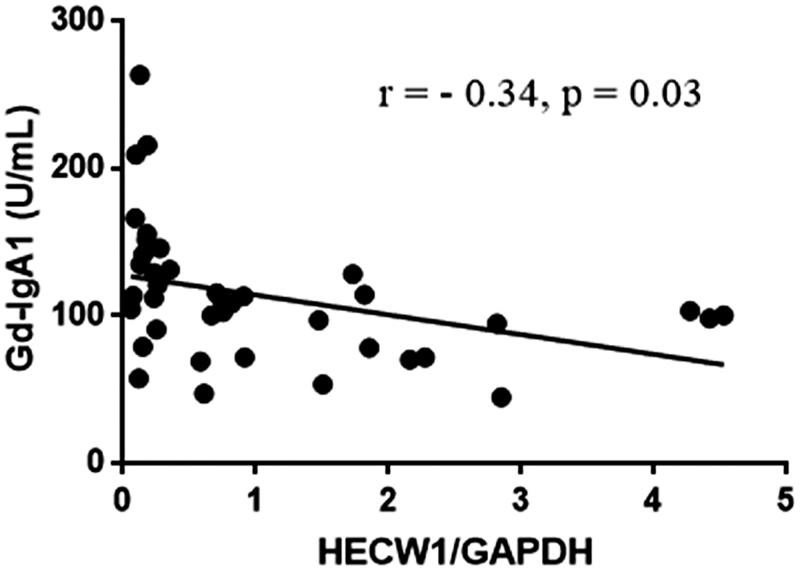
The correlation between HECW1 and Gd-IgA1 levels.

The levels of Gd-IgA1 (111.28 U/ml, [81.83 U/ml, 144.56 U/ml]) in IgAN patients were significantly higher than those of healthy controls (86.09 U/ml, [51.03 U/ml, 145.79 U/ml], *p* < .001; Figure 2). We further detected the association between HECW1 expression and Gd-IgA1 levels. Our data showed that the levels of Gd-IgA1 were significantly higher in low HECW1 level group compared with those in the high HECW1 level group (*p* = .02, Figure 3). And Gd-IgA1 levels were inversely correlated to the mRNA levels of HECW1 in IgAN (*r*= −0.34, *p* = .03, Figure 4).

### Association between rs978056 genotypes and HECW1 expression levels

**Figure 5.**
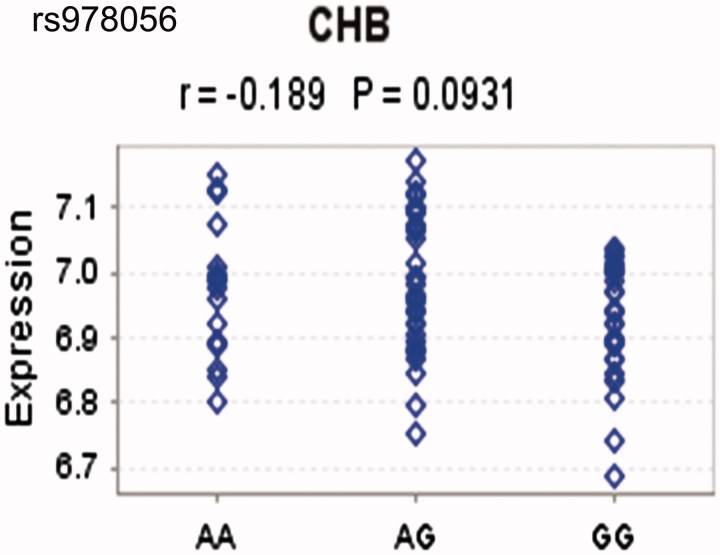
The correlation between rs978056 genotypes and HECW1 levels.

Since rs978056 located in the regulatory region, and hold the possibility to influence HECW1 expression. Association between rs978056 genotypes and HECW1 expression levels was further checked in healthy HapMap samples. Although non-significant association was observed between genotype of rs978056 and HECW1 mRNA expression in transformed B-cell lines, it seemed that the risk genotype (rs978056 GG) was associated with reduced HECW1 expression in 80 Han Chinese from Beijing, although the difference was not significant (CHB, *p* = .09, Figure 5).

### Clinical and pathological manifestation stratified by median level of HECW1

The differences of clinical and pathological manifestation were tested between the patients with high and low levels of HECW1. We found that plasma IgA1 levels were higher in low HECW1 levels group than those in high HECW1 levels group (*p* = .04, Table 3). No differences in age, gender, blood pressure, proteinuria, eGFR, uric acid, and Oxford pathological classification were demonstrated between these two groups (Table 3).

**Table 3.** Clinical and pathological manifestations of IgAN patients grouped according to HECW1 levels.

Variables	Low HECW1 Group (≤0.58, *n* = 20)	High HECW1 Group (>0.58, *n* = 20)	*p* value
Age (mean ± SD, year)	35.8 ± 9.76	38.11 ± 11.65	.88
Gender (M/F, %)	17 (60)/18 (40)	27 (50)/20 (50)	.50
SBP (mmHg, mean ± SD)	126 ± 13	125 ± 11	.67
Proteinuria (g/24h, median, IQR)	1.12 (0.45–3.80)	1.25 (0.43–3.54)	.45
eGFR (mL/min/1.73 m^2^, mean ± SD)	83.88 ± 30.40	80.21 ± 22.78	.83
Uric acid (μmol/L, mean ± SD)	411.23 ± 109.14	398.21 ± 80.44	.54
Plasma IgA1 (μg/mL, median, IQR)	1728 (980–2558)	1215 (765–1583)	.04
Oxford classification (%)			
M score (M0/M1)	2 (10)/18 (90)	4 (20)/16 (80)	.38
E score (E0/E1)	15 (75)/5 (25)	13 (65)/7 (35)	.49
S score (S0/S1)	9 (45)/11 (55)	13 (65)/7 (35)	.20
T score (T0/T1/T2)	10 (50)/8 (40)/2 (10)	8 (40)/10 (50)/2 (10)	.80
C score (C0/C1/C2)	3 (15)/14 (70)/3 (15)	7 (10)/8 (75)/5 (15)	.15

eGFR: estimated glomerular filtration rate; IQR: interquartile range; SBP: systolic blood pressure; SD: standard deviation.

## Discussion

Recent advances implicated that the overproduction of an aberrant form of IgA1 plays a key role in the pathogenesis of IgAN. Delineating the control of Gd-IgA1 production is important in understanding GN. Following previous GWAS that an allele localized to the *HECW1* gene was associated with Gd-IgA1 levels, we revealed for the first time the risk genotype (rs978056 GG) was associated with lowest HECW1 expression in Han Chinese from Beijing. Additionally, HECW1 mRNA levels were negatively correlated with Gd-IgA1 levels. These results suggest that there are many complicated factors responsible for altered IgA1 O-glycosylation besides dysregulated expression of glycosyltransferases.

Abnormalities in the O-glycan synthesis have been thought to be involved in several human diseases, including IgAN, inflammatory bowel disease (IBD), and cancers. However, the exact molecular perturbations that lead to O-glycosylation defects are presently unknown. Most studies conducted in IgAN populations identified *C1GALT1* and/or *C1GALT1C1* genes that are strongly associated with levels of Gd-IgA1; however, which only explains approximately 2%–7% of the variability in Gd-IgA1 levels, suggesting other factors are important in determining Gd-IgA1 levels in individuals [11,15]. In the current study, although no difference was found between IgAN and controls in the relative expression of HECW1, we found increased plasma levels of Gd-IgA1 in low HECW1 level group compared with those in high HECW1 level group. Furthermore, Gd-IgA1 levels had an inverse correlation with the mRNA levels of HECW1 in IgAN. Many studies addressing the functional significances associated with individual risk alleles could provide a more precise assessment of its relationship with disease. SNP-eQTL analysis supported that risk genotypes of rs978056 correlated with decreased HECW1 expressions, although the correlation did not reach statistical significance. No significant differences of clinical and pathological manifestation were observed between patients with high and low levels of HECW1. Our data supported that HECW1 involved in the production of galactose-deficient circulating IgA1 in IgAN patients.

HECW1 is primarily detected in human neuronal tissues and initially thought to regulate the bone morphogenetic protein signaling pathway during embryonic development and bone remodeling [16]. The ubiquitin-proteasome system is fundamental and important in recognition and degradation of misfolded proteins. HECW1 was found to ubiquitinate familial amyotrophic lateral sclerosis (FALS)-linked superoxide dismutase-1 gene for degradation which might contribute to the pathogenesis of FALS [13]. Li et al found HECW1 could target ErbB4 expression, a member of the epidermal growth factor receptor family, for ubiquitin-mediated degradation in breast cancer [17]. A dysregulated C1GALT1 enzyme is known to be involved in production of Gd-IgA1; the basis for its decreased function in IgAN is still unknown. Kiryluk et al showed HECW1 was a second-degree neighbor of C1GALT1 and COSMC, based on the analysis of known protein-protein interactions [11]. However, it is presently unknown, whether this protein participates in the proteosomal degradation of C1GALT1 resulting in the production of Gd-IgA1. Future studies will be needed to explore the relationship between HECW1 and C1GALT1 and its mode of action in B cells.

Recently, HECW1 protein was found in relatively low levels in neuroblastoma and breast cancer compared with normal tissue [17]. *In vitro* study, small interfering RNA-mediated knockdown of endogenous HECW1 could rescue HeLa cells from apoptosis [12]. HECW1 could cooperate with p53 to enhance its transcriptional pro-apoptotic activity, which may be capable of inducing apoptosis in cancerous cells possessing wild-type p53 [12,18]. The results of these previous studies indicate that HECW1 may act as a tumor suppressor gene in cancer. IgAN patients always carry a higher frequency of B cells in peripheral blood and the concentration of serum IgA is positively correlated with the frequency of B lymphocyte, which suggests the defects in the regulation of B cells survival [19]. In the following study, we found that, the low HECW1 levels were associated with high IgA1 levels; therefore, we suspected that decreased expression of HECW1 was due to mutations or external depression, which affects the survival of B lymphocytes and leads to increased production of galactose-deficient circulating IgA1.

A limitation of this study is the lack of clear evidence of association between HECW1 expression and IgAN susceptibility. The reason may be the underpowered sample size. Other ones may be mixed lymphocyte population in blood sample and possible environment or therapy influence. Additionally, the relationship between Gd-IgA levels and HECW1 expression are warranted to be further determined in more patients.

## Conclusion

In conclusion, we reported for the first time that there was an inverse correlation between HECW1 mRNA expression with Gd-IgA1 levels. Our study points to a new regulatory mechanism of IgAN that can explain the aberrant glycosylation of IgA1 responsible for the pathogenesis of the disease.

## Data Availability

Raw data used during the current study are available from the corresponding author on reasonable request for noncommercial use.

## Abbreviations

IgAN: IgA nephropathy
Gd-IgA1: Galactose-defcient IgA1
GWAS: Genome-wide association study
ESRD: End-stage renal disease
SNPs: Single nucleotide polymorphisms
eGFR: Estimated glomerular fltration rate
SBP: Systolic blood pressure
PBMC: Peripheral blood mononuclear cell
PBS: phosphatebuffered saline
BSA: bovine serum albumin
